# Vasorelaxant Effect of a New Hydrogen Sulfide-Nitric Oxide Conjugated Donor in Isolated Rat Aortic Rings through cGMP Pathway

**DOI:** 10.1155/2016/7075682

**Published:** 2015-11-09

**Authors:** Dan Wu, Qingxun Hu, Fenfen Ma, Yi Zhun Zhu

**Affiliations:** ^1^Department of Pharmacology, School of Pharmacy, Fudan University, Shanghai 201203, China; ^2^Department of Pharmacology, Loo Yong Lin School of Medicine, National University of Singapore, Singapore

## Abstract

Endothelium-dependent vasorelaxant injury leads to a lot of cardiovascular diseases. Both hydrogen sulfide (H_2_S) and nitric oxide (NO) are gasotransmitters, which play a critical role in regulating vascular tone. However, the interaction between H_2_S and NO in vasorelaxation is still unclear. ZYZ-803 was a novel H_2_S and NO conjugated donor developed by H_2_S-releasing moiety (*S*-propyl-L-cysteine (SPRC)) and NO-releasing moiety (furoxan). ZYZ-803 could time- and dose-dependently relax the sustained contraction induced by PE in rat aortic rings, with potencies of 1.5- to 100-fold greater than that of furoxan and SPRC. Inhibition of the generations of H_2_S and NO with respective inhibitors abolished the vasorelaxant effect of ZYZ-803. ZYZ-803 increased cGMP level and the activity of vasodilator stimulated phosphoprotein (VASP) in aortic rings, and those effects could be suppressed by the inhibitory generation of H_2_S and NO. Both the inhibitor of protein kinase G (KT5823) and the inhibitor of K_ATP_ channel (glibenclamide) suppressed the vasorelaxant effect of ZYZ-803. Our results demonstrated that H_2_S and NO generation from ZYZ-803 cooperatively regulated vascular tone through cGMP pathway, which indicated that ZYZ-803 had therapeutic potential in cardiovascular diseases.

## 1. Introduction

Due to the important role in cellular signal transduction, hydrogen sulfide (H_2_S) and nitric oxide (NO) are identified as gaseous transmitters. In vascular tissue, NO is synthesized from L-arginine by nitric oxide synthase (NOS) and it is considered to be the endothelium-derived relaxing factor. Evidence showed that in hypertensive patients the NO generation in endothelium cells was damaged [1]. Moreover, NO could prevent platelet activation and promote vascular smooth muscle cells proliferation [2]. H_2_S has been considered to be toxic gas for decades. But increasing study indicated that H_2_S could be protective against apoptosis and oxidative stress in cardiomyocytes [3]. H_2_S is synthesized from L-cysteine by cystathionine-*γ*-lyase (CSE) in vascular tissue [4]. Early study showed that H_2_S was a vasoactive factor and it could relax rat aorta through K_ATP_ channels in vascular smooth muscle cells [5]. H_2_S also had endothelium-independent vasorelaxation via vascular smooth muscle cells voltage-gated Ca^2+^ channels [6].

However, the interaction between H_2_S and NO is still controversial. Some researchers found that H_2_S could directly inhibit eNOS activity to suppress NO synthesis in rat and mouse aortic rings [7]. Study also showed that the deletion of H_2_S caused the increase of iNOS mRNA and protein expression levels in mice [8]. However, NO could enhance H_2_S generation, and H_2_S also could increase iNOS expression level under interleukin-1*β* (IL-1*β*) stimulation in vascular tissue [5, 9]. Coletta et al. reported that H_2_S and NO are mutually dependent to regulate endothelium-dependent vasorelaxation [10]. On the other hand, Whiteman et al. [11] and Ali et al. [12] found that the vasorelaxant effect of NO was inhibited by H_2_S.

Hypertension is caused by persistently high arteriotony, and it is a risk for the development of coronary heart disease, cerebrovascular diseases, heart failure, and so on [13]. Both H_2_S and NO can induce vascular relaxation to protect against hypertension; however the interaction effect and mechanism between them are still controversial. In this study, we synthesise a new slow-releasing H_2_S-NO donor, termed as ZYZ-803, to investigate the cross talk between H_2_S and NO in vasorelaxation effect.

## 2. Methods and Materials

### 2.1. Chemical Synthesis

Both 2-amino-3-prop-ynylsulfanyl-propionic acid (10 mmol) and Boc anhydride (10 mmol) were dissolved, subsequently stirred in tetrahydrofuran, eventually saturated with NaHCO_3_ (60 mL) for 5 h. The solution was then diluted with EtOAc (15 mL) and H_2_O (15 mL). The pH was also adjusted to 2–2.5 and then separate organic extracts were washed with brine and dried over Na_2_SO_4_. The crude material was purified by silica gel chromatography to give 2-(N-Boc-amino)-3-prop-2-ynylsulfanyl-propionic acid as oleaginous solid. Furoxan oxide was made as described previously [14]. Briefly, saturated aqueous sodium nitrite (217 mmol) was added into a solution of cinnamyl alcohol (75 mmol) in glacial acetic acid (15 mL) and then stirred for 1 h. The solution was diluted with H_2_O (15 mL) and extracted with diethyl ether (20 mL) for four times. The separate organic extracts were washed with brine and dried over MgSO_4_. The crude material was purified by silica gel chromatography to give furoxan as oleaginous solid. 2-(N-Boc-amino)-3-prop-2-ynylsulfanyl-propionic acid (0.6 mmol), furoxan oxide (0.6 mmol), N,N′-diisopropylcarbodiimide (0.6 mmol), and DPTS (0.6 mmol) were dissolved and stirred in DCM (50 mL) at room temperature for 5 h. The reaction mixture was diluted with DCM and washed three times with H_2_O (15 mL). The organic layer was separated and dried over Na_2_SO_4_ (60 mL) and then solvent was removed by rotary evaporation. The crude product was eluted by 5% MeOH/PE from silica gel. The final product was oleaginous solid and verified by ^1^H nuclear magnetic resonance spectroscopy.

### 2.2. Chemicals and Antibodies

The following antibodies were used: anti-vasodilator stimulated phosphoprotein (VASP) antibody, anti-p-VASP antibody, anti-eNOS antibody, and anti-p-eNOS antibody were purchased from Cell Signaling Technology, and anti-CSE and anti-*β*-actin-antibody were purchased from Santa Cruz.

All drugs were purchased from Sigma unless otherwise stated.

### 2.3. Animals

Male Sprague-Dawley (SD) rats (6-7 weeks old) were maintained on standard conditions and were free to receive food and water. Animals were handled according to the Guide for the Care and Use of Laboratory Animals, published by the US National. Experimental procedures were managed according to the local ethical committee of Fudan University.

### 2.4. Isolated Rat Aortic Ring Assay

SD rats (8–10 weeks of age) were anesthetized by isoflurane inhalation and dissected to obtain thoracic aortas, which were subsequently cleaned from connective tissue. Rings (2-3 mm in length) were cut and placed in organ bath (10 mL) filled with oxygenated (95% O_2_ to 5% CO_2_) Krebs-Henseleit solution at 37°C, mounted to isometric force transducers, and connected to a data acquisition system. The aortic rings were allowed to equilibrate at a basal tension of 2 g for 1 h. Subsequently rings were contracted with phenylephrine (PE; 1 *μ*mol/L) until a plateau was reached. In order to identify the integrity of the endothelium, an acetylcholine (Ach) was operated on PE-contracted rings. Rings that relaxed less than 80% were abandoned, unless the removal of endothelium was specified. 60 min later, a series of experiments 1–5 were performed. As least five aortic rings were carried out for each experiment, which were from different rats.

#### 2.4.1. Series 1

This series of experiments were performed to determine the effects of cumulative concentrations (1–100 *μ*mol/L) of *S*-propyl-L-cysteine (SPRC), furoxan, SPRC + furoxan, and ZYZ-803 on the sustained contractile response to PE (1 *μ*mol/L) in endothelium-contained and endothelium-uncontained aortic rings.

#### 2.4.2. Series 2

This series of experiments were performed to determine the time course (1–60 min) of 100 *μ*mol/L SPRC, furoxan, SPRC + furoxan, and ZYZ-803 on the sustained contractile response to PE (1 *μ*mol/L) in endothelium-intact aortic rings.

#### 2.4.3. Series 3

In this series of experiments, the effects of cumulative concentrations (1–100 *μ*mol/L) of ZYZ-803 on the sustained contractile response to PE (1 *μ*mol/L) were detected in endothelium-intact aortic rings. In order to investigate the role of endogenous H_2_S and NO generations in PE-contracted aortic rings, the endothelium-intact aortic rings were incubated with PAG (1 mmol/L) and L-NAME (50 *μ*mol/L) for 60 min, respectively.

#### 2.4.4. Series 4

This series of experiments were performed to investigate whether K_ATP_ channels played a key role in the vasorelaxant effect of cumulative concentrations (1–100 *μ*mol/L) ZYZ-803 on PE-induced (1 *μ*mol/L) contraction. The endothelium-intact aortic rings were incubated with glibenclamide (10 *μ*mol/L) for 60 min.

#### 2.4.5. Series 5

In this series, the vasorelaxant effect of cumulative concentrations (1–100 *μ*mol/L) ZYZ-803 on PE-induced (1 *μ*mol/L) contraction was studied under the inhibition of the activity of VASP. The endothelium-intact aortic rings were incubated with KT5823 (1 *μ*mol/L) for 60 min.

### 2.5. Detection of H_2_S and NO Level

Aortic rings were incubated with 100 *μ*mol/L SPRC, furoxan, or ZYZ-803 for 1 h in organ bath filled with oxygenated (95% O_2_ to 5% CO_2_) Krebs-Henseleit solution at 37°C. Rings were homogenized and subsequently centrifuged (12000 r/min, 15 min) and eventually the supernatant was collected. The H_2_S level in aortic rings was measured as descripted previously [15]. 75 *μ*L homogenized aortic ring matrix was mixed with 250 *μ*L zinc acetate (1%, w/v), 425 *μ*L distilled water, 133 *μ*L *N*-dimethyl-*p*-phenylenediamine sulfate (20 mmol/L, 7.2 mmol/L HCl), and 133 *μ*L FeCl_3_ (30 mmol/L, 1.2 mmol/L HCl) for 10 min incubation at room temperature. 250 *μ*L trichloroacetic acid (10%) was added and then centrifuged at 14,000 r/min, 5 min. The absorbance of samples at 670 nm was detected by the microplate reader (Infinite 1000, Tecan Systems Inc.). The NO level in aortic rings was measured with Griess Assay Kit (Beyotime Institute of Biotechnology) according to the manufacturer's protocol.

### 2.6. Western Blot Analysis

Aortic rings were homogenized with homogenization buffer (Beyotime Institute of Biotechnology). And western blot analysis was used to determine the protein expression level as previously described [3]. The images were captured by Image System (Bio-Rad) with an enhanced chemiluminescence detection kit (Millipore). Protein concentration was determined by BCA assay kit (Pierce).

### 2.7. Detection of Cyclic GMP (cGMP) Level

The cGMP level in aortic rings was measured with cGMP ELISA kit (R&D Systems) according to the manufacturer's protocol.

### 2.8. Statistical Analysis

All data show mean ± SEM, statistical analysis was performed using one-way ANOVA, and post hoc pairwise comparisons were performed using Prism graph. A *p* value < 0.05 was considered significant difference.

## 3. Results

### 3.1. The Vasorelaxant Effect of ZYZ-803 on the Sustained Contraction Induced by PE

**Figure 1:**
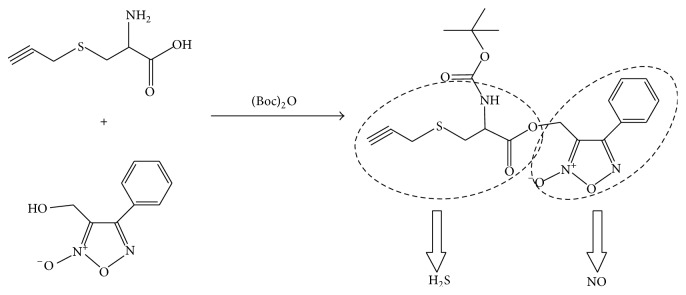
Chemical synthesis of ZYZ-803. Boc: *t*-butyloxycarbonyl.

**Figure 2:**
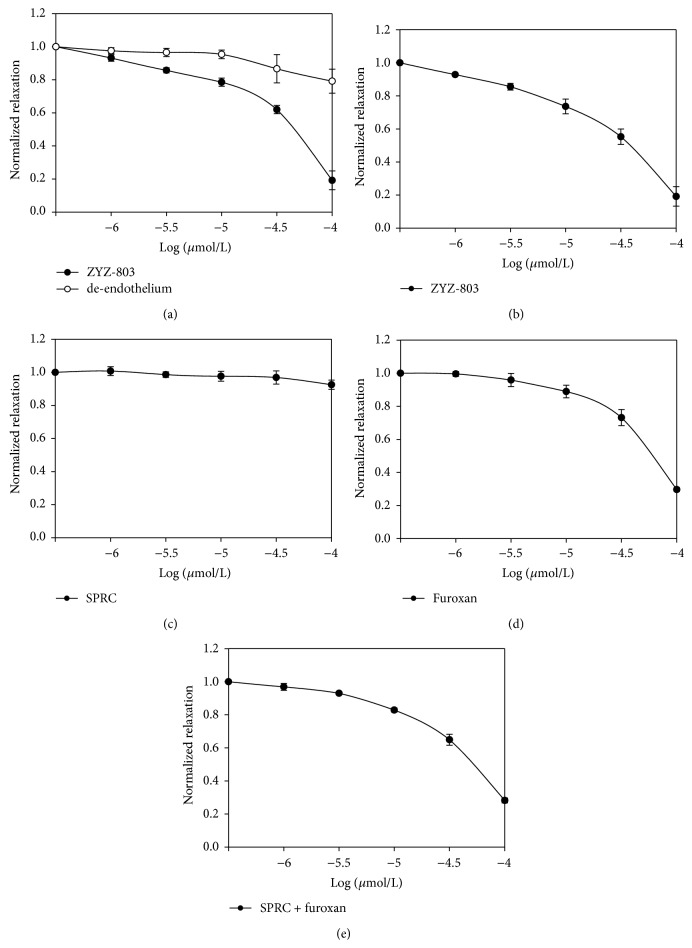
ZYZ-803 could dose-dependently relax the sustained contraction induced by PE in aortic rings. (a) The vasorelaxant effect of ZYZ-803 on the sustained contraction induced by PE was endothelium-dependent. The vasorelaxant effect of ZYZ-803 (b), the vasorelaxant effect of SPRC (c), the vasorelaxant effect of furoxan (d), and the vasorelaxant effect of the mixture of SPRC and furoxan (e) on the sustained contraction induced by PE in endothelium-contained aortic rings. Data represent mean ± SEM, *n* ≥ 5 for each group.

SPRC, synthesized by our group, has been proved that it could increase H_2_S concentration through promoting CSE expression [16]. Furoxan is a NO donor, and it can release NO in the presence of thiols [14]. In this study, we made SPRC react with *t*-butyloxycarbonyl (Boc) anhydride and then coupled it with furoxan by ester bond to develop a new H_2_S-NO conjugated donor, termed as ZYZ-803 (Figure 1). In endothelium-uncontained aortic rings, ZYZ-803 could not significantly relax the PE-induced contraction (Figure 2(a)). However, exposure of endothelium-contained aortic rings to ZYZ-803, at 1–100 *μ*mol/L, caused a significant and concentration-dependent relaxation (Figure 2(b)). It indicated that the vasorelaxant effect of ZYZ-803 was endothelium-dependent. And at a concentration of 100 *μ*mol/L, ZYZ-803 caused maximal relaxation that was about 85%. After treatment with SPRC or furoxan, the vasorelaxant response curve was shifted to the ZYZ-803 dose-dependent curve right (Figures 2(c)-2(d)). And, at the same vasorelaxant effect, ZYZ-803 was at least 100-fold or 1.5-fold more potent than SPRC or furoxan, respectively. Treatment with the mixture of SPRC and furoxan also caused less vasorelaxant effect than that of treatment with ZYZ-803 in PE-contracted endothelium-contained aortic rings (Figure 2(e)).

### 3.2. ZYZ-803 Stimulated Vasorelaxation Slowly

**Figure 3:**
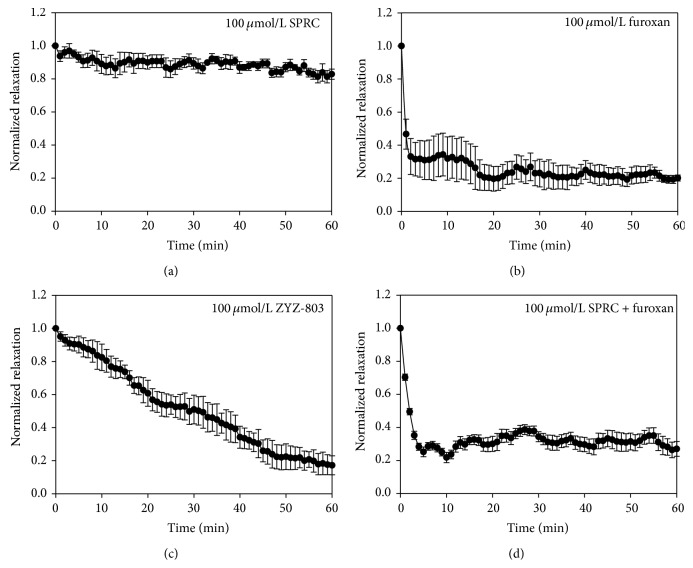
The time course of vasorelaxant effects of testing compounds in aortic rings on the sustained contraction induced by PE. (a) The vasorelaxation time-effect curve of 100 *μ*mol/L SPRC. (b) The vasorelaxation time-effect curve of 100 *μ*mol/L furoxan. (c) The vasorelaxation time-effect curve of 100 *μ*mol/L ZYZ-803. (d) The vasorelaxation time-effect curve of 100 *μ*mol/L furoxan plus 100 *μ*mol/L SPRC. Data represent mean ± SEM, *n* ≥ 5 for each group.

To investigate the vasodilator rate, we measured the time course of vasorelaxant effects of testing compounds. At 100 *μ*mol/L, SPRC did not cause significant relaxation of PE-contracted endothelium-contained aortic rings (Figure 3(a)). At the same concentration, furoxan could induce the relaxation effect of endothelium-contained aortic rings significantly under sustained contraction induced by PE (Figure 3(b)). However, this vasorelaxant effect was so rapid and unstable. Treatment with 100 *μ*mol/L ZYZ-803 caused remarkable and time-dependent vasorelaxant effect in PE-induced contracted aortic rings. In the meanwhile, the vasorelaxant response to ZYZ-803 was much slower and sustained long residual action than that to furoxan (Figure 3(c)). Interestingly, the mixture of SPRC and furoxan only induced rapid vasorelaxant effect, which was unlike the effect of ZYZ-803 (Figure 3(d)).

### 3.3. Vasorelaxant Effect of ZYZ-803 on PE-Induced Contractions after Inhibition of CSE and/or eNOS

**Figure 4:**
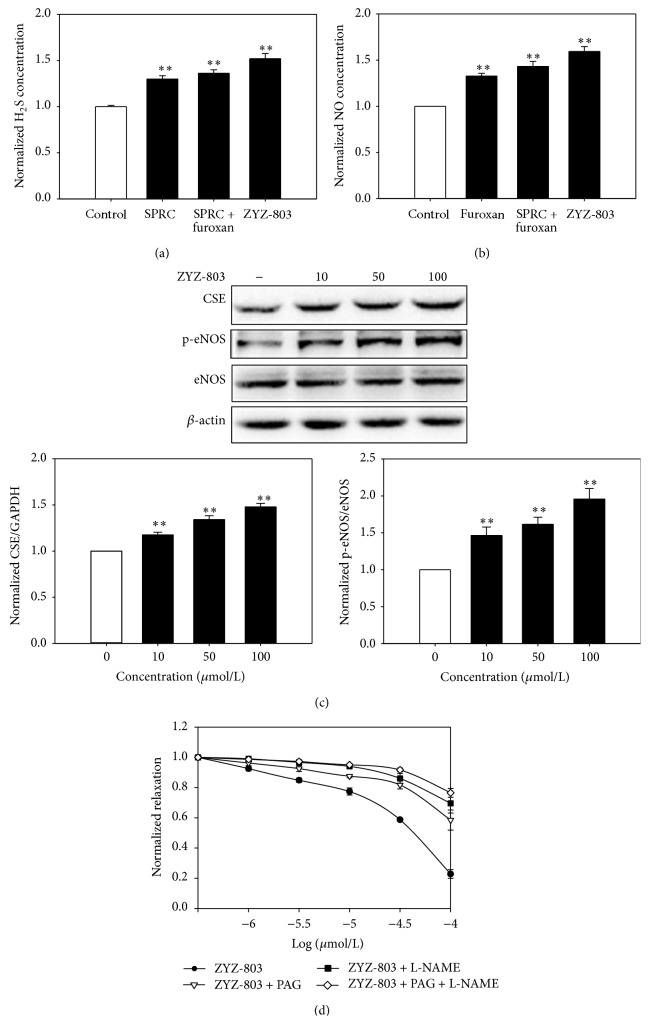
Vasorelaxant effect of ZYZ-803 on PE-induced contractions was suppressed upon inhibition of endogenous H_2_S or NO generation. (a) The concentration of H_2_S in aortic rings after SPRC, SPRC + furoxan, and ZYZ-803 treatments. (b) The concentration of NO in aortic rings after furoxan, SPRC + furoxan, and ZYZ-803 treatments. (c) The expressions of CSE, eNOS, and p-eNOS after ZYZ-803 (10, 50, and 100 *μ*mol/L) treatments. (d) The vasorelaxant effect of ZYZ-803 after L-NAME, PAG, and L-NAME + PAG treatments on PE-induced contractions in aortic rings. Data represent mean ± SEM, *n* ≥ 5 for each group. ^*∗∗*^ *p* < 0.01 compared with control group.

All of SPRC, SPRC + furoxan, and ZYZ-803 could induce the generation of H_2_S in aortic rings. In the meantime, the H_2_S level in ZYZ-803 treatment was the highest one among these three treatments (Figure 4(a)). A similar result was observed in the level of NO in aortic rings. ZYZ-803 caused more generation of NO than SPRC + furoxan or furoxan alone (Figure 4(b)). As shown in Figure 4(c), ZYZ-803 could increase CSE expression and eNOS activity dose-dependently. Considering that H_2_S and NO had good vasorelaxant effects, the further experiment was to identify the interaction between H_2_S and NO. As shown in Figure 4(d), both CSE inhibitor PAG and eNOS inhibitor L-NAME, as well as the mixture of PAG and L-NAME, could suppress the vasorelaxant of ZYZ-803. And the inhibitory vasorelaxation of PAG + L-NAME was more severe. It indicated that both H_2_S and NO played a key role, and these two gases were mutually promoted in the regulation of vascular tone.

### 3.4. Vasorelaxant Effect of ZYZ-803 on PE-Induced Contractions after Inhibition of K_ATP_ Channel

**Figure 5:**
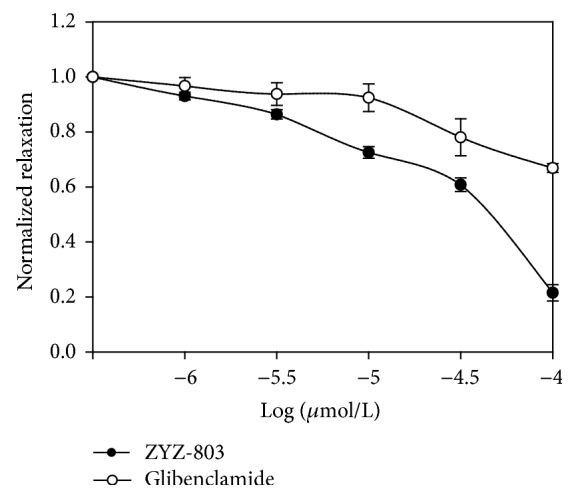
Vasorelaxant effect of ZYZ-803 on PE-induced contractions was suppressed after inhibition of K_ATP_ channel. The vasorelaxant effect of ZYZ-803 was abolished on PE-induced contractions in aortic rings after glibenclamide treatment. Data represent mean ± SEM, *n* ≥ 5 for each group.

Early studies indicated that H_2_S and NO could regulate vascular tone through opening K_ATP_ channel [5, 17, 18]. In order to assess the role of K_ATP_ channel in the vasorelaxant effect of ZYZ-803, we did the test in PE-contracted endothelium-contained aortic rings that were pretreated with glibenclamide, a K_ATP_ channel inhibitor. As shown in Figure 5, 1–100 *μ*mol/L ZYZ-803 relaxed the contraction evoked by PE in a dose-dependent manner. However, the vasorelaxant response to ZYZ-803 was significantly abolished in the presence of glibenclamide. This result demonstrated that ZYZ-803 was a K_ATP_ channel opener to relax vascular to.

### 3.5. The Vasorelaxant Effect of ZYZ-803 through cGMP Pathway

**Figure 6:**
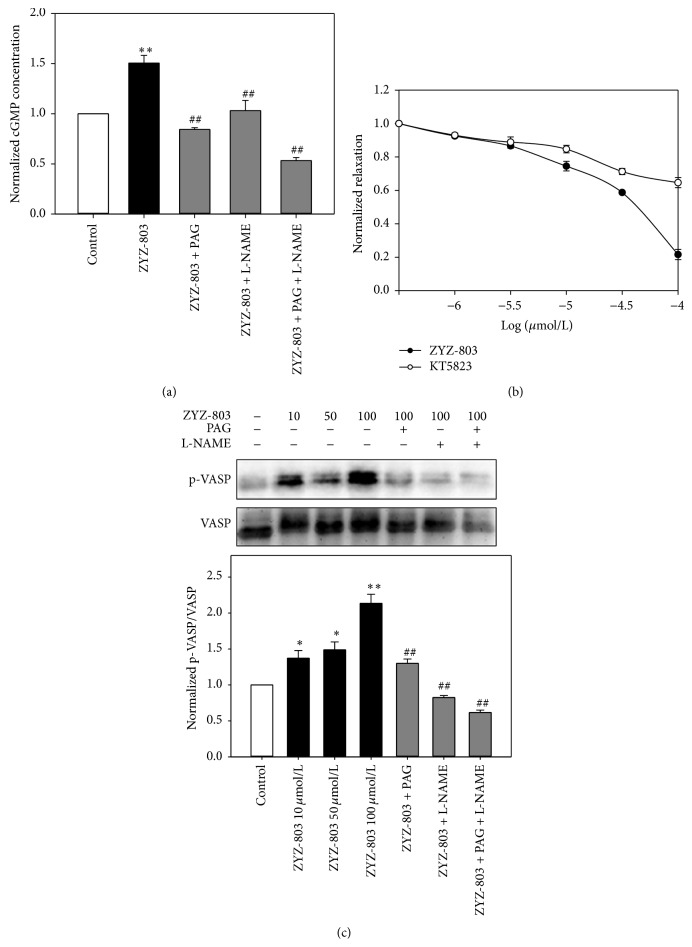
ZYZ-803 relaxed aortic rings on PE-induced contractions through cGMP pathway. (a) The ZYZ-803-induced cGMP increase was abolished after L-NAME, PAG, and L-NAME + PAG treatments. (b) The vasorelaxant effect of ZYZ-803 on PE-induced contractions was suppressed after KT5823 treatment in aortic rings. (c) The expression level of VASP and p-VASP in aortic rings. Data represent mean ± SEM, *n* ≥ 5 for each group. ^*∗∗*^ *p* < 0.01 compared with control group. ^##^ *p* < 0.01 compared with ZYZ-803 group.

cGMP was considered to be one of the second messengers that regulate vascular tone under physiological conditions. The cellular level of cGMP is the balance of synthesis and degradation. cGMP is synthesized by soluble guanylyl cyclase (sGC) [19]. Previous study had shown that NaHS could time- and dose-dependently increase cGMP level in rat aortic smooth muscle cells [20], and NO could also increase cGMP level in mice aortic rings [21]. Considering the critical role of cGMP in vasorelaxation, we studied whether there was any effect of ZYZ-803 on cGMP concentration. As shown in Figure 6(a), the level of cGMP was elevated by ZYZ-803 treatment, whereas this effect was attenuated by PAG and/or L-NAME treatment. cGMP can activate its downstream signaling molecule protein kinase G (PKG). We found that the vasorelaxant effect of ZYZ-803 was inhibited when PKG inhibitor KT5823 was used in PE-induced contraction aortic rings (Figure 6(b)). VASP serine-239 is the major phosphorylation site of PKG, and it was used as the marker of PKG activity. In aortic rings, treatment with ZYZ-803 dose-dependently increased the phosphorylation level of VASP at serine-239 site, and PAG and/or L-NAME could inhibit VASP activity (Figure 6(c)). The inhibitory effects of PAG + L-NAME on cGMP level and VASP activity were more severe than that of PAG or L-NAME alone. These results indicated the cooperation of H_2_S and NO on cGMP/VASP pathway in vascular tissue.

## 4. Discussion

Endothelium-dependent vasorelaxation injury is known to lead many cardiovascular diseases, such as hypertension, atherosclerosis, and heart failure [19]. NO generation from eNOS is considered to be endothelium-derived relaxing factor [21]. Like NO, H_2_S is an endogenous gaseous transmitter and it also can regulate vascular tone under physiological and pathological conditions [22]. Growing evidence has shown that the interactions between H_2_S and NO play an important role in vasorelaxation; however the precise nature of the interaction is still unclear. Furoxan is a NO donor that is used for NO bioactivity widely in preclinical studies [23]. SPRC is an effective endogenous H_2_S donor, which could increase CSE expression and activity to protect against ischemia reperfusion injury [16]. In the current study, we conjugated furoxan with SPRC to develop a novel releasing H_2_S and NO donor, termed ZYZ-803. We used ZYZ-803 to study the interaction between H_2_S and NO and investigated the therapeutic potential of ZYZ-803 for vasorelaxation.

Endothelial cells exist between blood vessels and blood, which is a single layer of cells. It can secrete a variety of bioactivator substances to regulate vascular functions, like vascular tone, blood pressure, blood coagulation, and migration of leukocytes. NO is secreted in endothelial cells to relax blood vessel through cGMP pathway. And NO donors have vasorelaxant effect to decrease blood pressure. It has been reported that the concentration of H_2_S in hypertension rats is lower than that in normal rats [24]. H_2_S caused a dose-dependent relaxation from preconstricted aortic rings through K_ATP_ channel [5]. Growing evidence has shown the interactions between H_2_S and NO play an important role in vasorelaxation. Hosoki et al. found that H_2_S and NO regulated vasorelaxation in the synergistic effect [25]. Nevertheless, Zhao and Wang reported that the vasorelaxant activity of NO was inhibited by H_2_S [22]. NO reacted together with H_2_S to produce a new molecule that exhibited little vasorelaxant activity [12]. However, Eberhardt et al. showed that nitroxyl was generated by the chemical reaction of H_2_S and NO, which could stimulate vasorelaxation [26]. These results suggested that the complex cross talk between H_2_S and NO is of great importance. In this paper, we found that, at the same concentration, ZYZ-803 had more competitive vasorelaxant effect compared with SPRC and furoxan alone. This result indicated that H_2_S and NO could mutually potentiate relaxant effect of one another in aortic rings.

In this study we found that SPRC, furoxan, and ZYZ-803 showed different time-effective curve in vasorelaxation. 100 *μ*mol/L SPRC did not show a significant relaxation in aortic rings. At the same concentration, the vasorelaxant effect of furoxan was effective and rapid. However, a rapid relaxation of blood vessels leads to a range of side effects, such as vascular damage, low blood pressure, and rapid heart rate. 100 *μ*mol/L ZYZ-803 relaxed aortic rings stably, enduringly, and more effectively than furoxan. Several studies suggested that the effective concentration of H_2_S to relax vessels was high, usually in 300 *μ*mol/L–1 mmol/L [27–29]. SPRC was linked to furoxan by an ester bond. With the ester bond dissociated slowly, SPRC and furoxan could stimulate the generations of gasotransmitters. Therefore, different from the mixture of SPRC and furoxan, ZYZ-803 could generate H_2_S and NO over a long time period and performed more significant than SPRC + furoxan in vasorelaxant effect. Indeed, ZYZ-803 may be a good strategic treatment for vascular chronic diseases.* In vivo*, the generation of gasotransmitters is a slow progression and their concentrations are low under physiological condition. Initial studies demonstrated that some donors released a large amount of H_2_S or NO during a short time, which caused a variety of side effects and increased the toxicity of these donors [30, 31]. Furthermore, in previous studies, the doses of gasotransmitter donors were high in diseases treatments due to the effumability of gases. ZYZ-803 released H_2_S and NO slowly and lasted for a long time, which were close to the physiological environment. Compared with treatment of SPRC and furoxan alone, treatment of ZYZ-803 had fewer side effects with lower concentration. So ZYZ-803 will be a useful tool for biological researches.

In vascular tissue, H_2_S is generated from smooth muscle cells, endothelial cells, and perivascular adipose cells by CSE. And NO is generated by eNOS in endothelial cells [19]. Furoxan could increase the level of H_2_S induced by SPRC, and SPRC could increase the level of NO induced by furoxan. Moreover, the H_2_S and NO levels of treatment with ZYZ-803 were higher than those of SPRC and/or furoxan. We speculated that the reason was that ZYZ-803 could keep concentrations of H_2_S and NO in the effective dose range for a long time, which could stimulate CSE and eNOS to generate more H_2_S and NO, respectively. ZYZ-803-induced vasorelaxation was abolished by L-NAME and/or PAG, which indicated that ZYZ-803 produced H_2_S and NO through the stimulation of CSE and eNOS, which had synergetic effect on vasorelaxation.

It has been shown that both NO and H_2_S are K_ATP_ channel openers in vascular tissue [5, 19]. In this study, we found that pretreatment with glibenclamide, the inhibitor of K_ATP_ channel, suppressed ZYZ-803-induced vasorelaxation. This result demonstrated that ZYZ-803 is a novel K_ATP_ channel opener in rat aortic rings. cGMP/PKG pathway plays a great role in the endothelium-dependent vasorelaxation. The second messenger cGMP can activate PKG and then induced K_ATP_ channel open. The ratio of p-VASP/VASP is a sensitive monitor to detect the cGMP/PKG pathway signaling [32]. Studies have shown that NO can activate sGC and increases intracellular cGMP level in vascular tissue [19]. Like NO, treatment with H_2_S time- and concentration-dependently increases cGMP level in rat aortic smooth muscle cells [33]. In this study, ZYZ-803 could improve cGMP level and phosphorylation level of VASP in aortic rings. And these effects could be abolished by treatment with L-NAME and/or PAG, which indicated that H_2_S and NO were mutually dependent on the activation of cGMP pathway.

## 5. Conclusions

In summary, we synthesized a novel H_2_S-NO conjugated donor, ZYZ-803, which exhibited more potential in vasorelaxation compared with the parent drugs in aortic rings. ZYZ-803 opened K_ATP_ channel through cGMP pathway and exerted an effective, stable, and durable vasorelaxation. H_2_S and NO could improve the generation of one another and created synergism in the regulation of vascular tone. ZYZ-803 could be very useful to study the interaction between H_2_S and NO better in different physiological and pathological conditions, and it would open a new prospect in researches of gasotransmitters. In addition, ZYZ-803 exhibits powerful therapeutic potential in vasorelaxation, which sheds new light on further gaseous drugs design for the treatment of cardiovascular diseases.
